# Cancer module genes ranking using kernelized score functions

**DOI:** 10.1186/1471-2105-13-S14-S3

**Published:** 2012-09-07

**Authors:** Matteo Re, Giorgio Valentini

**Affiliations:** 1 Dipartimento di Informatica, Università degli Studi di Milano, via Comelico 39/41, 20135 Milano MI, Italia

## Abstract

**Background:**

Co-expression based Cancer Modules (CMs) are sets of genes that act in concert to carry out specific functions in different cancer types, and are constructed by exploiting gene expression profiles related to specific clinical conditions or expression signatures associated to specific processes altered in cancer. Unfortunately, genes involved in cancer are not always detectable using only expression signatures or co-expressed sets of genes, and in principle other types of functional interactions should be exploited to obtain a comprehensive picture of the molecular mechanisms underlying the onset and progression of cancer.

**Results:**

We propose a novel semi-supervised method to rank genes with respect to CMs using networks constructed from different sources of functional information, not limited to gene expression data. It exploits on the one hand local learning strategies through score functions that extend the guilt-by-association approach, and on the other hand global learning strategies through graph kernels embedded in the score functions, able to take into account the overall topology of the network. The proposed kernelized score functions compare favorably with other state-of-the-art semi-supervised machine learning methods for gene ranking in biological networks and scales well with the number of genes, thus allowing fast processing of very large gene networks.

**Conclusions:**

The modular nature of kernelized score functions provides an algorithmic scheme from which different gene ranking algorithms can be derived, and the results show that using integrated functional networks we can successfully predict CMs defined mainly through expression signatures obtained from gene expression data profiling. A preliminary analysis of top ranked "false positive" genes shows that our approach could be in perspective applied to discover novel genes involved in the onset and progression of tumors related to specific CMs.

## Background

Large scale projects aimed at the elucidation of the molecular mechanisms underlying tumors onset and progression play a crucial role to improve clinicians ability to treat cancer [1]. The huge amount of data produced by these research projects yielded to the development of specialized data repositories enabling researchers to mine effectively cancer expression related data like ONCOMINE [2], and to collect and organize information about the gene expression profiles of normal, pre-cancer, and cancer cells as in the case of the Cancer Genome Anatomy Project (CGAP). Cancer specific gene expression data can also be found in the Gene Expression Omnibus (GEO) repository [3]. The availability of this unprecedented volume of data has, on the one hand, the potential to boost the research focused on the elucidation of the molecular basis of cancer and, on the other hand, to accelerate the development of novel cancer therapies.

Even if novel bio-technologies, such as Next Generation Sequencing and epigenetic pattern analysis, have been recently applied to cancer research [4], a fundamental contribution in this research area is still due to the application of gene expression profiling. This technique proved to be effective for the classification of diverse types of tumors [5], for the prediction of patients outcome [6] and the prediction of the response to chemotherapies [7,8].

By exploiting gene expression profiling, Segal and colleagues constructed a functional module map for cancers to investigate commonalities and variations between different types of tumor [9]. The novelty of their approach lies in the analysis of expression profiles for the identification of sets of genes that act in concert to carry out specific functions in different cancer types, and in the construction of a module map constituted by a collection of the gene sets associated to specific Cancer gene Modules (CMs, hereafter). The rationale behind this approach is that the comparison of molecular profiles can reveal both the existence of specific patterns (represented in this case by the expression profiles) and the biological behavior of distinct tumor types, without the need to integrate other sources of information, such as gene regulatory networks or molecular pathways, known to be relevant for the molecular characterization of cancer.

Despite the identification of Cancer Modules based on a single type of molecular evidence reduces the complexity of the problem, this approach introduces also serious limitations. Indeed the CMs are identified considering only transcriptional signatures, but it is commonly accepted that some of the aberrations leading to cancer onset and driving their progression do not occur at transcription level [10]. A second and more important limitation regards the interpretability of CMs: being derived from transcriptional data only, the functional interpretation of the CMs cannot be easily translated into a wider biological context, since other molecular processes, ranging from post-transcriptional to translational and post-translational events may finely regulate the final product of genes. For instance, gene transcripts must be translated into proteins by the ribosomes and misregulations of this important process can contribute to several diseases, including cancer [11-13].

As a consequence, gene expression data alone, even if fundamental to identify CMs, cannot detect genes involved, for instance, in post-transcriptional misregulated processes underlying cancer. To this end we need other sources of data (i.e. protein-protein interactions, metabolomic data and many others) to confirm CMs identified mainly through transcriptional data, and to discover novel genes, not detectable with gene expression profiling, related to the molecular pathology of tumors.

In this contribution we test the hypothesis that the CMs published in [9] can be predicted through network-based algorithms using different sources of functional interaction data, not limited to correlations between expression profiles. To this end we integrated functional interaction networks derived from Reactome and other curated databases, and from uncurated pairwise relationships (e.g. protein-protein and protein domain-domain interactions), from protein complexes and from comparative genomics techniques [14,15]. Moreover we propose a novel algorithm to rank genes with respect to their potential membership to each specific CM. The different ranking methods proposed in the literature in general exploit local or global learning strategies to properly rank genes/nodes in a biomolecular network [16-19]. In this paper we propose a ranking method that combines both local and global learning strategies to exploit both "local" similarities between genes and "global" similarities embedded in the topology of the network. Indeed our proposed *kernelized score functions *adopt both local learning strategies based on a generalized notion of distance in a universal reproducing kernel Hilbert space, and global learning strategies based on the choice of proper graph kernels to exploit the overall topology of the underlying biological network. Moreover our proposed approach is modular and extensible, in the sense that different variants of both local and global learning strategies can be chosen to design different gene ranking algorithms. Our networks-based algorithms are not only able to recover the CMs by using functional networks resulting from different sources of biomolecular data, but in perspective they could be also applied to discover novel genes involved in the onset and progression of tumors related to specific CMs.

## Methods

In this section at first we describe the Cancer gene Modules (CMs) proposed in [9] and the functional interaction networks used in our experiments to rank genes according to their likelihood to belong to specific CMs. Then we propose a fast semi-supervised machine learning method based on kernelized score functions to rank genes with respect to Cancer Modules: the proposed approach adopts both local and global learning strategies able to exploit different notions of functional similarity between genes and the overall functional relationships between genes encoded in the topology of the network. We also briefly summarize two state-of-art semi-supervised machine learning methods for node ranking in biomolecular networks, i.e. the *GeneMANIA *[18], and the *LabProp *algorithms [17], and finally we introduce the integration techniques adopted to combine the functional interaction networks.

All the methods described below process an undirected weighted graph *G *= <*V*, *E *>, where *V *is the set of vertices representing genes and *E *the set of edges representing functional similarity between pairs of genes. For the sake of simplicity we denote with *v *∈ *V *both a vertex of the graph and the corresponding associated gene. ***W ***is the corresponding adjacency matrix with elements *w_ij _*representing the "strength" of the similarity between vertices *v_i_*, *v_j _*∈ *V*, and *V_C _*⊂ *V *is a subset of genes belonging to a given Cancer Module.

### Cancer gene modules

The CMs [9] were obtained from the Molecular Signatures Database, MSigDB [20] (class: **C4 **(computational gene sets), set name: **CM **Cancer Modules). In [9] Segal and colleagues investigated the expression profiles of 14145 genes in 1975 arrays spanning 17 clinical categories represented by several types of tumour. To this end the authors collected 2849 publicly available gene sets and identified the arrays in which each gene set shows an expression signature (coordinated over or under expression) of a consistent part of the genes belonging to the considered gene set. Problems due to consistent overlaps between the signatures associated to different gene sets were solved by clustering the gene sets on the basis of their core signatures. This led to the definition of 456 statistically significant gene sets called modules by the authors (see [9] for further details). In the second step of their analysis the authors used these modules to characterize clinical conditions associated to the arrays, according to the combination of modules that are activated and deactivated. This work has the merit to be among the first that tried to investigate commonalities and variations between different types of tumour in terms of sets of altered functional gene modules.

### Functional interactions networks

In this section we describe the functional gene networks used in our tests and the reasons motivating their usage with respect to the prediction of the CMs identified in [9]. We used both protein-protein and domain-domain interaction networks enforced through the predictions of a classifier [14], and functional interaction networks constructed with comparative genomics techniques [15].

#### Computationally predicted functional protein interaction network

In [14] Wu and colleagues constructed a functional protein interaction network (*FI*) based on functional interactions predicted by a Naive Bayes classifier (NBC) trained on pairwise relationships extracted from Reactome [21] and other curated pathways databases, and from uncurated pairwise relationships derived from physical protein-protein interactions (PPI) in human and other species, from gene co-expression data, proteins domain-domain interactions, protein interactions obtained via biomedical text mining, and Gene Ontology annotations. The constructed network was then applied to the study of several types of tumors (with a focus on Glioblastoma multiforme).

The rationale behind this approach is that the usage of a classifier able to predict the occurrence of a true functional interaction (which is not directly implied by the observation of a PPI) can be exploited in the construction of a functional interaction network that combines high-coverage unreliable pairwise interactions datasets with low-coverage highly reliable pathway-based functional interactions. This network was used in our experiments because the classifier trained on many and diverse datasets can embed in the predicted functional interaction links not only information derived from human gene co-expression data but also from protein-protein and protein domain-domain interactions.

#### Comparative genomics based enrichment of functional interaction networks

Similar in spirit to the approach in [14], the functional network construction method presented in [15] by Lee and colleagues integrates diverse lines of evidence in order to produce a functional human gene network (*HumanNet*) that has then been used in several tests to predict causal genes for human diseases and to increase the power of genome-wide association studies. *HumanNet *and *FI *networks include different sources of functional interaction evidences: e.g. protein domain-domain interactions data are not involved in the construction of *HumanNet *and data about protein complexes are not considered in the construction of the *FI *network.

The most significant difference between the two networks consists in the inclusion in *HumanNet *of functional interactions borrowed from other species through comparative genomics techniques: functional interactions have been propagated from yeast, fly and worm to human by means of a comparative genomics approach presented in [22,23] and previously validated in other species [24,25].

### Score functions based on kernelized similarity measures

**Figure 1:**
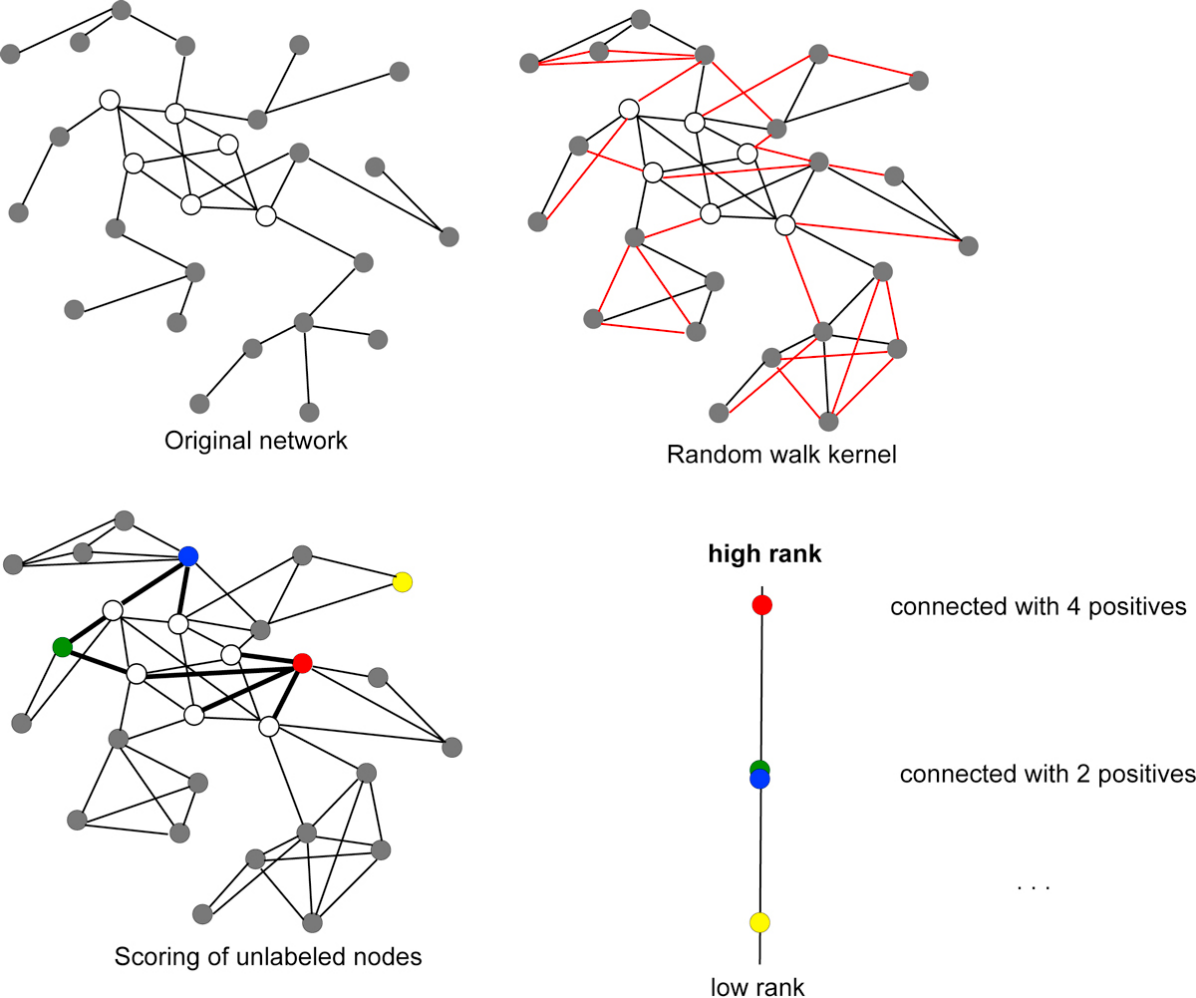
**Overview of the main logical steps of the proposed method**. I) Top left: the original graph representing functional interactions between genes. White nodes correspond to labeled examples (genes belonging to a given CM), gray nodes to unlabeled examples. II) Top right: the "augmented" graph obtained by applying a 2-step random walk kernel. Red edges represent the new connections between genes added by the random walk kernel. III) Bottom left: genes scoring. The score function is applied to 4 coloured nodes: the weights of the edges (outlined in boldface) connecting the coloured nodes to the labeled "positive" nodes are added to obtain the scores associated to each coloured node. IV) Bottom right: gene ranking. The coloured nodes are ranked according to the previously computed scores.

Kernelized score functions are based on: a) score functions that generalize the guilt-by-association approach [16,26] by introducing different functions to quantify the similarity between a gene and its neighbours in a biomolecular network; b) an extended notion of similarity between genes implemented through kernels embedded in the score functions. The approach is modular, in the sense that the score functions are designed for general kernels, and specific kernels can be applied or specifically designed to represent similarities between genes connected in functional networks. The proposed algorithm is fast and scales well with large functional networks. A schematic overview of the proposed procedure is depicted in Figure 1.

More precisely, by this approach we can derive score functions *S: V *→ ℝ^+ ^based on properly chosen kernel functions, by which we can directly rank vertices according to the values of *S*(*v*): the higher the score, the higher the likelihood that a gene belongs to a given Cancer Module. From this standpoint our approach is related to, and can be considered an extension of a method recently proposed in the different context of gene function prediction from synthetic lethality networks [27].

The score functions are built on distance measures defined in a suitable Hilbert space ℋ and computed using the usual "kernel trick", by which instead of explicitly computing the inner product *< φ *(·), *φ *(·) > in the Hilbert space, with *φ: V *→ ℋ, we compute the associated kernel function *K: V *× *V *→ ℝ^+ ^in the original input space *V *. Let be *D*(*v*, *V_C_*) a suitable distance measure in the Hilbert space between a given vertex/gene *v *and the set of genes *V_C _*belonging to a specific Cancer Module. We chose three different distance measures:

(1) $$D_{AV}\left(v,\;V_{C}\right)=\frac{1}{{|V}_{C}|}\underset{x\in V_{C}}{\sum }||\;\phi \left(v\right)-\phi \left(x\right)||{}^{2}$$

(2) $$D_{NN}\left(v,\;V_{C}\right)=\underset{x\in V_{C}}{\text{min}}||\phi \left(v\right)-\phi \left(x\right)||{}^{2}$$

(3) $$D_{kNN}\left(v,\;V_{C}\right)=\underset{x\in I_{k}\left(v\right)}{\sum }||\phi \left(v\right)-\phi \left(x\right)||{}^{2}$$

where *I_k_*(*v*) in (3) represents the first ranked *k *vertices *x *∈ *V_C _*according to *K*(*v*, *x*). These distances represent respectively the *average*, the *nearest-neighbors *and the *k-nearest-neighbors *distance in ℋ of the vertex *v *w.r.t. the set of vertices *V_C_*. From these distances we can derive three score measures, respectively the *Average score*, the *Nearest Neighbours *and the *K-Nearest Neighbours *scores.

**Average score**. By developing the square in (1) we obtain:

(4) $$D_{AV}\left(v,\;V_{C}\right)=<\phi \left(v\right),\phi \left(v\right)>+\frac{1}{\left|V_{C}\right|}\underset{x\in V_{C}}{\sum }<\;\phi \left(x\right),\phi \left(x\right)>-\frac{2}{\left|V_{C}\right|}\underset{x\in V_{C}}{\sum }<\phi \left(v\right),\phi \left(x\right)>$$

By recalling that <*φ*(·), *φ*(·) *>*= *K*(·,·), to obtain a similarity measure we need only to change the sign of (4):

(5) $$Sim_{AV}\left(v,\;V_{C}\right)=-K\left(v,\;v\right)+\frac{2}{\left|V_{C}\right|}\underset{x\in V_{C}}{\sum }K\left(v,\;x\right)-\frac{1}{\left|V_{C}\right|}\underset{x\in V_{C}}{\sum }K\left(x,\;x\right)$$

By observing that the third term of (5) is equal for all *v *∈ *V *, we can obtain the following *Average score S_AV_*:

(6) $$S_{AV}\left(v,\;V_{C}\right)=-K\left(v,\;v\right)+\frac{2}{\left|V_{C}\right|}\underset{x\in V_{C}}{\sum }K\left(v,\;x\right)$$

Note that if all *K*(*v*, *v*) are equal for all *v*, we can further simplify (6) by removing its first term.

**Nearest-neighbours score**. If instead of considering the average distance (1) between a vertex *v *and *V_C_*, we consider the minimum distance between *v *and *V_C _*in the feature space (2), we can derive in a similar way the similarity measure *Sim_NN_*:

(7) $$Sim_{NN}\left(v,\;V_{C}\right)=-\underset{x\in V_{C}}{\text{min}}\;\left[K\left(v,\;v\right)-2K\left(v,\;x\right)+K\left(x,\;x\right)\right]$$

If *K*(*x*, *x*) is equal for all *x *∈ *V*, we can simplify (7), thus achieving the *nearest neighbours score S_NN_*:

(8) $$S_{NN}\left(v,\;V_{C}\right)=-\underset{x\in V_{C}}{\text{min}}-2K\left(v,\;x\right)=2\underset{x\in V_{C}}{\text{ max }}K\left(v,\;x\right)$$

**K-nearest-neighbours score**. A natural extension of the *S_NN _*score can be derived from the *k-nearest neighbours distance *(3) of a vertex *v *from the set of nodes *V_C_*, thus obtaining the *k-nearest neighbours score S_kNN_*:

(9) $$S_{kNN}\left(v,\;V_{C}\right)=2\underset{x\in I_{k}\left(v\right)}{\sum }K\left(v,\;x\right)$$

Any valid kernel *K *can be applied to compute the above proposed scores, but in the context of Cancer Module gene ranking, we used *random walk kernels *[28], since they can capture the similarity between genes, taking into account the topology of the overall functional interaction network. Given a symmetric adjacency matrix ***W ***of the functional interaction undirected graph *G*, the *one-step random walk kernel *is:

(10) $$K=\left(a-1\right)I+D^{-\frac{1}{2}}WD^{-\frac{1}{2}}$$

where ***K ***is the Gram matrix associated to the random walk kernel function, whose elements *k_ij _*correspond to the values *K*(*v_i_*, *v_j_*) of the kernel function, ***I ***is the identity matrix, ***D ***is a diagonal matrix with elements $d_{ii}=\sum_{j}w_{ij}$, and *a *is a value larger than 1.

In our experiments we applied *q-step random walk kernels **K **_q-step _*= ***K***^*q*^, by varying the number of steps *q *[28]. In this way we can explicitly evaluate the direct neighbors of each gene (*q *= 1), but also its undirected neighbors (e.g. *q *= 2 or *q *= 3). In other words, by setting *q *= 2 or *q *= 3 two vertices are considered similar if they are directly connected or if they are connected through a path including one or two vertices. In principle also longer paths could be considered, but this could introduce too remote similarities between genes, yielding a potential high level of noise in the prediction of Cancer Module genes.

It is worth noting that Vavien, a recently proposed method applied to the gene ranking problem with respect to OMIM diseases using protein-protein interaction networks [29], shares some ideas, but also shows significant differences with our approach. The general setting of the problem is similar, but the realization of the score function is very different: the Vavien algorithm proposes a simple correlation measure between topological profiles and the average profiles of genes known to belong to a specific OMIM class, while we propose different score functions, based on different notions of distance, and the average distance that resembles the Vavien average profile is realized in a more general Hilbert space, and represents only one of the possible distances that can be considered. From this standpoint our approach could be considered a generalization of Vavien: our method is not restricted to the classical correlation measure to model the similarity between genes, but different notions of similarity can be realized through the proper choice of a kernel function: the correlation can be applied by using a correlation kernel [30], but other kernels representing different notions of similarity between genes, (e.g. graph kernels [28] able to capture the overall topology of the network), can be embedded in the score functions to rank genes.

### GeneMANIA

*GeneMANIA *[18] is a variant of the semi-supervised learning algorithm originally proposed by Zhou et al. [31], by which, adopting a "Gaussian smoothing" approach labels associated to the vertices can be propagated to rank the unlabeled vertices of the network. Similarly to the previous method, *GeneMANIA *finds a score *S*(*v_i_*) for each *vi *∈ *V *, according to their likelihood to belong to a given class *V_C_*, by minimizing the following objective function:

(11) $$S^{*}=\text{arg}\;\underset{S}{\text{min }}\alpha \underset{i}{\sum }{\left(s_{i}-s_{i}^{0}\right)}^{2}+\left(1-\alpha \right)\underset{i}{\sum }\underset{j}{\sum }w_{ij}{\left(s_{i}-s_{j}\right)}^{2}$$

where ***S ***is the vector of the scores associated to the genes, ***S***^0 ^is the initial vector of scores reflecting the a priori knowledge about the investigated genes, *s_i _*and $s_{i}^{0}$ their *i^th ^*components, and *w_ij _*are the elements of the adjacency matrix ***W ***of the graph *G *connecting the genes. Note that eq. (11) is the convex combination (0 ≤ *α *≤ 1) of two terms, where the first one minimizes the error between predicted and a priori known scores, while the second assures the "internal coherence" of the network, by penalizing connected genes (i.e. pairs of genes *v_i _*and *v_j _*with *w_ij _*> 0) having different scores. Equation (11) can be solved in closed form or through efficient iterative algorithms (e.g. error minimization by conjugate gradient techniques). *GeneMANIA*, originally proposed to predict gene functions, differs from the original Zhou algorithm since it introduces a simple but effective cost-sensitive technique (useful when the number of positive examples is largely lower than the total number examples), and moreover applies a novel weighted integration technique [32] (see "Networks integration" below).

### Label propagation (LabelProp)

The Zhu et al. *LabelProp *(Label Propagation) [17] algorithm minimizes an objective function that resembles the previously described Zhou et al. algorithm:

(12) $$S^{*}=\text{arg}\;\underset{S}{\text{min}}\underset{i}{\sum }\underset{j}{\sum }w_{ij}{\left(s_{i}-s_{j}\right)}^{2}$$

Eq. (12) corresponds to the second summation of eq. (11), that assures an "internal coherence" of the computed score (see previous subsection). The coherence w.r.t. the initial score ***S***^0 ^is assured by not allowing any change of the scores *s_i _*for the vertices *v_i _*∈ *V_C _*during the label propagation process, that is the predicted scores *s_i _*are set to $s_{i}^{0}$ for each *v_i _*∈ *V_C_*. Also this algorithm can be implemented both in closed form, or through iterative techniques.

### Networks integration

To integrate the *FI *and the *HumanNet *networks, we summed their corresponding adjacency matrices, previously normalized according to a Laplacian graph normalization, thus assuring the symmetry of the resulting normalized matrix [28]. This method has been applied to integrate the data with all the methods, but with *GeneMANIA *we also used the *SW *algorithm, since it has been introduced as part of an enhanced version of *GeneMANIA *[32]. In brief, *SW *integrated the networks according to a weighted sum strategy, i.e. through a weighted sum of the corresponding adjacency matrices ***W***^(*i*)^:

(13) $$W^{*}=\underset{i}{\sum }w_{i}W^{\left(i\right)}$$

The weights *w_i _*are computed simultaneously for all the considered classes by solving efficiently a single ridge regression problem [32].

## Results and discussion

After introducing the general set-up of the experiments, we at first show that our proposed kernelized score functions can successfully rank genes with respect to CMs, using different sources of functional interaction data, i.e. the *FI *and *HumanNet *functional networks (see section "Functional interaction networks"), even if CMs are defined mainly in terms of over or underexpressed sets of genes. Then we compare our proposed kernelized score functions with several state-of-the-art network-based gene ranking methods, using both separated *FI *and *HumanNet *data and an integrated data set constructed by combining the two functional networks. Finally, we show that our methods could be applied to discover novel genes associated to specific cancer types, by analyzing whether top ranked "false positive" genes for the CM 234 (*Bone osteoblastic module*) are actually involved in the onset and progression of types of cancer related to CM 234.

### Experimental set-up

The genes belonging to the CMs defined in] [9] were filtered in order to ensure the presence of at least one functional interaction in both the *FI *and *HumanNet *networks (see Methods): this led to the definition of a final collection of 8499 human genes. We then removed each Cancer Module annotated with less than 20 genes, since our aim consists in assuring reliable predictions and in showing the feasibility of our approach, obtaining a final set of CMs composed of 298 distinct modules.

For each CM we ranked the genes with respect to their likelihood to belong to the core set of genes annotated to the considered module. Performance evaluation was realized following a canonical 5-folds stratified cross-validation (CV) scheme repeated 5 times. Performances were collected in terms of precision at fixed recall levels (ranging from 0.1 to 1.0 at 0.1 steps). We also computed the area under the ROC curve (AUC) for each CM. The results were averaged across the CV folds and the repetitions of the experiment. We finally registered the computational times required by each method for the completion of the entire experiment.

### Ranking of genes using multi-source functional interaction networks

**Figure 2:**
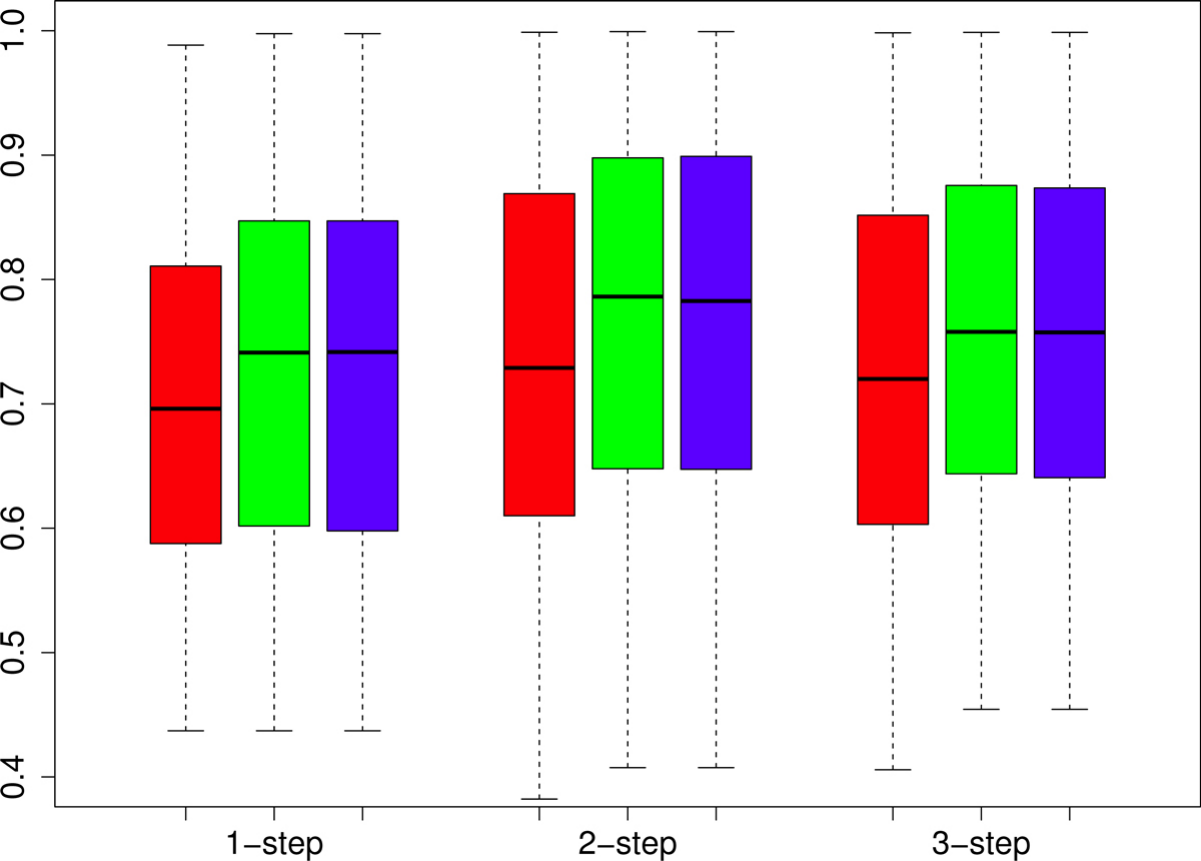
**Ranking of genes using the *HumanNet *functional interaction network: distribution of AUC results across the 298 Cancer modules**. From left to right boxplots refer to 1-step, 2-step and 3-step random walk kernels. Red boxplots correspond to *S_NN_*, green to *S_kNN _*and blue to *S_AV _*kernelized score functions.

**Figure 3:**
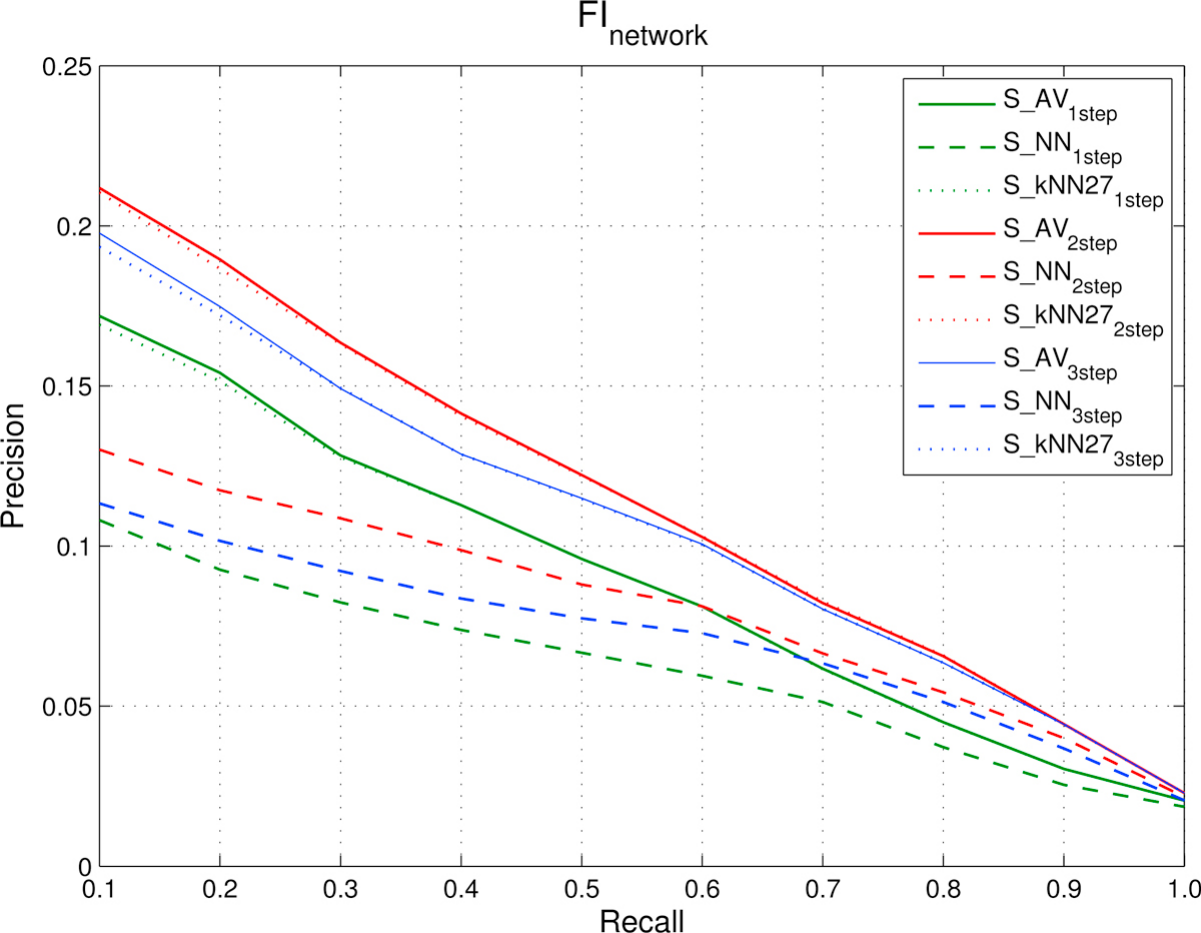
**Functional interactions network (*FI*): precision and recall curves relative to different kernelized score functions using random walk kernels at 1, 2 and 3 steps**. precisions, averaged across the 298 Cancer Modules, are computed through 5-fold cross-validation techniques repeated 5 times for different fixed recall levels ranging from 0.1 to 1. *S_AV _*stands for *Average score*, *S_NN _*for *Nearest-neighbor score *and *S_kNN _*for *k-Nearest-neighbor score*.

**Figure 4:**
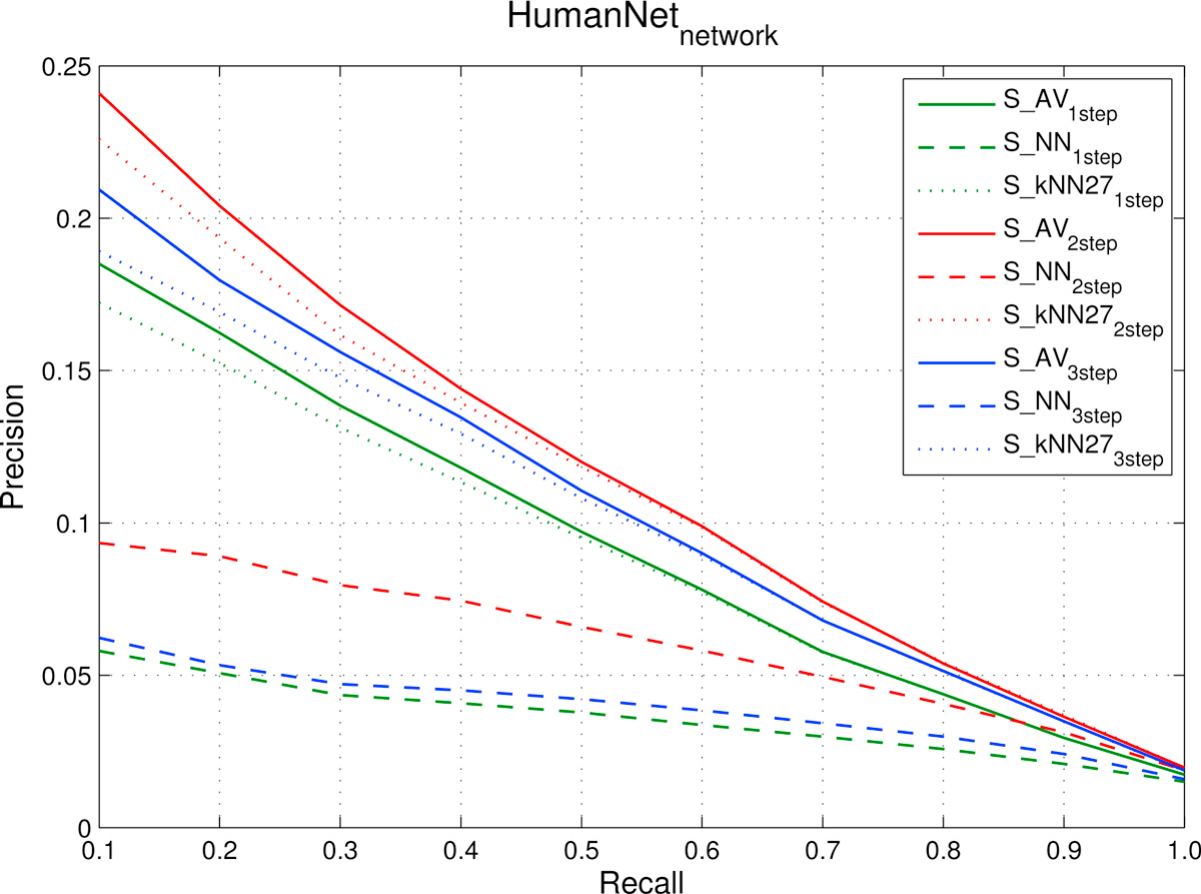
***HumanNet *network: precision and recall curves relative to different kernelized score functions using random walk kernels at 1, 2 and 3 steps**. precisions, averaged across the 298 Cancer Modules, are computed through 5-fold cross-validation techniques repeated 5 times for different fixed recall levels ranging from 0.1 to 1. *S_AV _*stands for *Average score*, *S_NN _*for *Nearest-neighbor score *and *S_kNN _*for *k-Nearest-neighbor score*.

We designed a set of experiments to show that CMs are predictable using sources of data not limited to gene expression profiles. More precisely our aim consists in showing that we can rank genes with respect to a specific cancer module using protein-protein or domain-domain interaction data included in the *FI *network, or by using other functional interaction data obtained through comparative genomics techniques as the ones included in the *HumanNet *networks (see Methods for more details on these networks). To this end, according to the experimental set-up described in the previous sections, we applied our newly proposed kernelized score functions *S_NN_*, *S_kNN _*and *S_AV_*, using 1, 2 and 3-steps random walk kernels. AUC results presented in Figure 2 show that the proposed methods are able to rank genes with respect to cancer modules using functional interaction networks constructed with different sources of biomolecular data: independently of the score function and the kernel adopted, the AUC values with *HumanNet *are always significantly larger than 0.5 for most of the 298 CMs. Similar results are obtained also with the *FI *functional network (data not shown). These results are also confirmed by the precision-recall curves averaged across the 298 CMs (Figure 3 and 4), that show that the kernelized score functions can reasonably learn the cancer modules using *FI *and *HumanNet *networks.

The proper choice of the optimal number of random walk steps for the kernelized score functions is of critical importance in order to obtain good performances. As we can see in Figure 3 and 4, independently of the choice of the kernelized score function and of the considered functional interaction network, the best performance in terms of precisions at fixed recall levels is obtained with 2-steps random walk kernels. AUC results show that 2-steps random walk kernels are the optimal choice also with respect to this metric (Figure 2). We thus decided to use only kernelized score functions based on 2-steps random walk kernel in the subsequent analyses. The choice of the optimal number of neighbours (the *k *parameter in *S_kNN_*) was tuned by internal cross validation. We repeated the entire experiment (using both the separated and integrated networks) by varying *k *between 3 and 29. By averaging across classes, we found that optimal average results (both in terms of precision and AUC) are obtained with *k *= 27. The *a *parameter of the kernel functions (Section "Score functions based on kernelized similarity measures") has been set to 2 for all the ranking tasks, after a preliminary evaluation of other values. It is worth noting that both the score functions (i.e. the type of score function) and the parameters of random walk kernels (i.e. the number of steps, the *a *parameter and the number of neighbours for *S_kNN_*) can be tuned e.g. by internal cross validation separately for each CM. This is a computationally intensive approach that could yield to better results, but in principle it could be feasible considering that the proposed kernelized score functions are very fast (see the section "Comparison of the empirical time complexity" below).

The results show that functional similarities encoded in interaction networks are thus useful to rank genes with respect to cancer modules. In particular, direct and indirect neighbours (coded respectively in 1 and 2-steps kernels) are on the average the most informative to correctly rank genes. Indeed 2-steps random walk kernels take into account both direct links and nodes with path length equal to 2 (indirect neighbours) to rank genes. If we include in the score evaluation also nodes with path length equal to 3 on the average we can observe a certain decay in performance. A larger decay is observed with 4-steps random walk kernels (data not shown). These results show that similarities mediated through direct common neighbours (2-steps) are the on the average the most informative to predict CMs. Loose similarities, represented by connections between genes mediated through two or more other genes may add noise to the learning process, thus resulting in reduced performance.

We need meaningful networks constructed with informative functional interactions between genes to correctly rank genes according to CMs. For instance, we hypothesize that simple GO annotations to construct similarity networks between genes are not enough to predict whether a gene may belong to a specific cancer module. To test this hypothesis we evaluated the performances obtained by ranking the genes using directly as input a network based on GO functional annotations shared between genes. Using *S_AV _*with a 2 steps random walk kernel we obtained an average precision close to 0:04 at recall 0.1, and this value decreases from recall 0:1 to 1:0 (data not shown). The poor performance obtained with networks constructed from GO annotations were also confirmed by AUC results, very close to 0.5, indicating, in practice, absence of learning. These results are consistent with the process of definition of the CMs, since even if many of them are composed of subsets of one or more gene sets corresponding to functional classes as encoded by GO or other functional annotations repositories, CM design policies require that all the members of the signatures constituting the core of a CM must be up or down regulated [9].

### Comparison of kernelized score functions with other gene ranking methods

We compared our proposed kernelized score functions *S_AV _*(*Average score*), *S_NN _*(*Nearest neighbors score*) and *S_kNN _*(*k-Nearest neighbors score*) (see "Methods") with other semi-supervised machine learning methods for gene ranking in biomolecular networks: *GeneMANIA *[18,32], the semi-supervised network-based method proposed by Zhou and others [33] (closely related to *GeneMANIA*), and the label propagation method (*LabelProp*), proposed in [17] (see "Methods"). Results are presented separately for the three functional interaction networks (*FI *network, *HumanNet *network and the integrated network).

#### Results using *FI *and *HumanNet *networks

**Figure 5:**
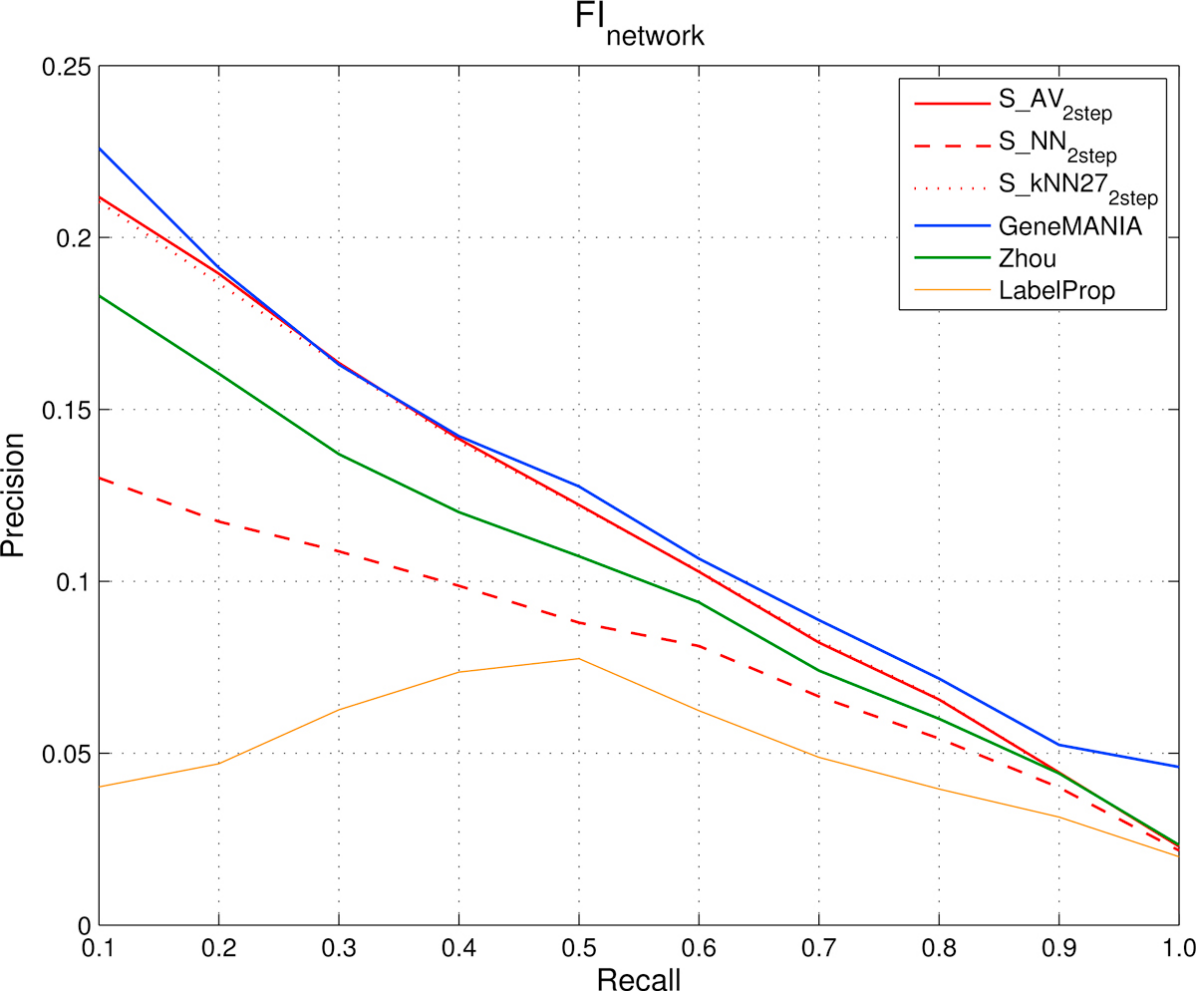
***FI *network: comparison of precision and recall curves between our proposed kernelized score functions and other machine learning methods for gene ranking**. precisions, averaged across the 298 Cancer Modules, are computed through 5-fold cross-validation techniques repeated 5 times for different fixed recall levels ranging from 0.1 to 1. *S_AV _*(*Average score*), *S_NN _*(*Nearest-neighbor score*) and *S_kNN _*(*k-Nearest-neighbor score*) represent kernelized score functions. The parameter *k *of *S_kNN _*is set to 27. *Zhou *is the algorithm based on Gaussian Random Fields proposed in [31] and *GeneMANIA *its variant, while *LabelProp *is the *Label Propagation *algorithm proposed in [17].

**Figure 6:**
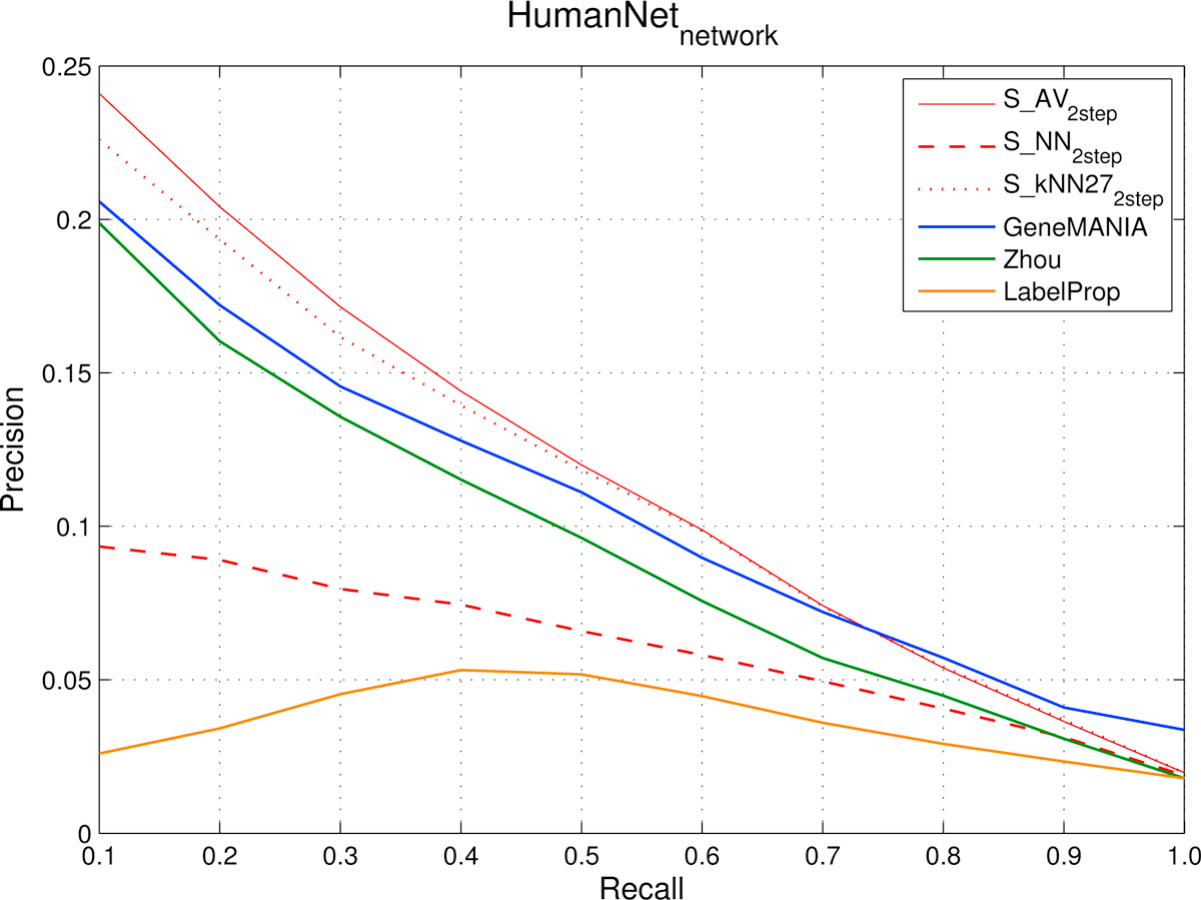
***HumanNet *network: comparison of precision and recall curves between our proposed kernelized score functions and other machine learning methods for gene ranking**. precisions, averaged across the 298 Cancer Modules, are computed through 5-fold cross-validation techniques repeated 5 times for different fixed recall levels ranging from 0.1 to 1. *S_AV _*(*Average score*), *S_NN _*(*Nearest-neighbor score*) and *S_kNN _*(*k-Nearest-neighbor score*) represent kernelized score functions. The parameter *k *of *S_kNN _*is set to 27. *Zhou *is the algorithm based on Gaussian Random Fields proposed in [31] and *GeneMANIA *its variant, while *LabelProp *is the *Label Propagation *algorithm proposed in [17].

Figure 5 (*FI *network) and Figure 6 (*HumanNet *network) show the compared results obtained by the different methods.

When using the functional relationships encoded in the *FI *network (Figure 5), *GeneMANIA *performs slightly better than all the other compared methods, with the exception of precisions from 0.2 to 0.4 recall levels, where results are very close to those obtained by *S_AV _*and *S_kNN_*. The *Zhou *method (of which *GeneMANIA *can be considered an enhanced version) performs worse than *GeneMANIA*, *S_AV _*and *S_kNN _*in terms of precision, but better than *S_NN_*. The worst performance in terms of precision was obtained by *LabelProp*. All the precision curves share the same trend (monotonically decreasing) with the exception of the curve of *LabelProp *which shows a maximum at recall 0.5.

When using the relationships encoded in the *HumanNet *network (Figure 6), the best precisions at recall ranging from 0.1 to 0.6 were obtained by *S_AV _*and *S_kNN _*while the best precision in the 0.8 to 1.0 recall range are obtained by *GeneMANIA*. The precisions of *S_AV_*, *S_kNN _*and *GeneMANIA *are constantly above the ones of the other methods. Also with this dataset *LabelProp *confirmed its poor performance: the main reasons of these results depend on both the nature of this algorithm and the characteristics of the functional interaction networks. Indeed *LabelProp *propagates the initial labeling to all the network by performing multiple iterations of the label propagation before to converge to a stable solution. In this way the algorithm explores also nodes very far from the core of the initial positive nodes, and genes are considered similar even when paths connecting them are relatively long; as a consequence, two genes become "similar" when their functional similarities are relatively loose, thus introducing noise in the transductive process of gene ranking with respect to the CMs.

#### Results using the integrated functional interaction network

**Figure 7:**
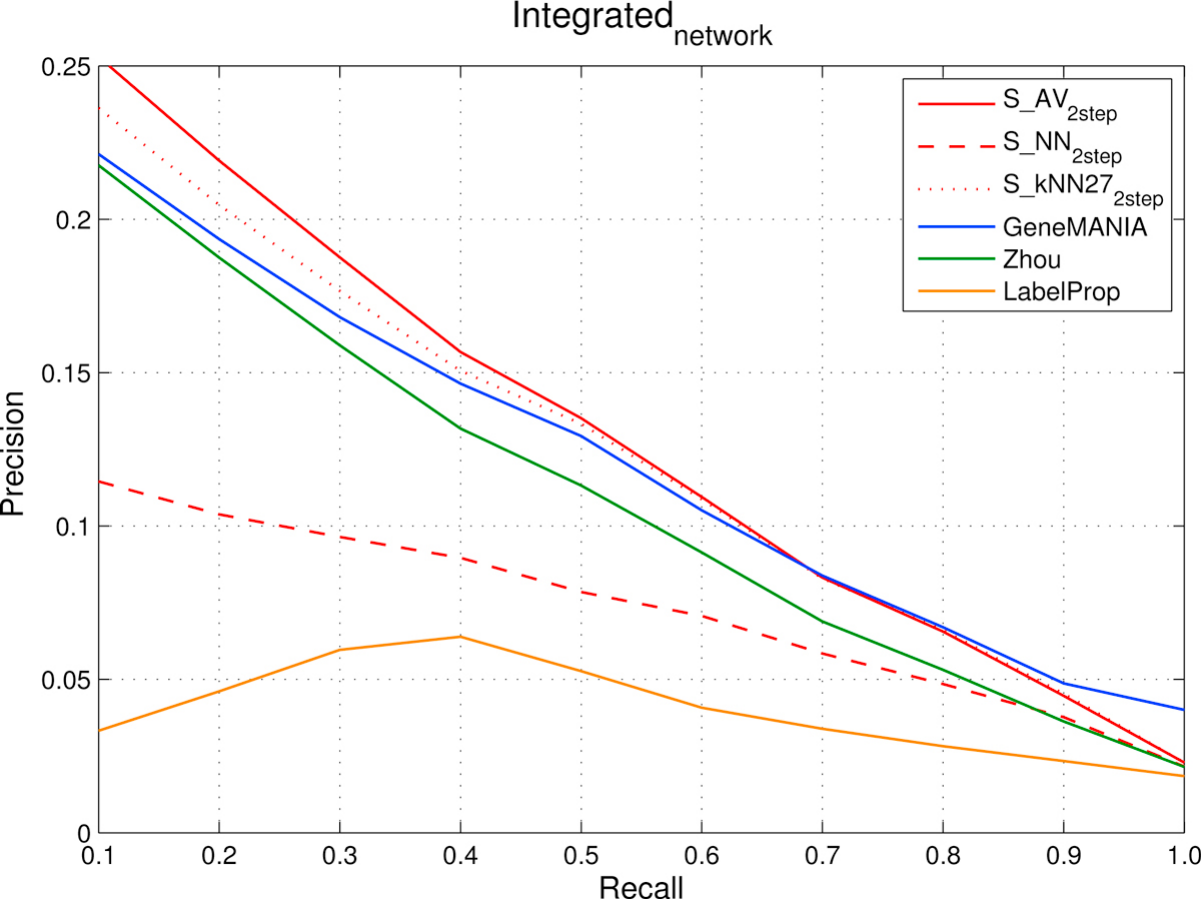
**Integrated network obtained by combining *FI *and *HumanNet *networks: comparison of precision and recall curves between our proposed kernelized score functions and other machine learning methods for gene ranking**. precisions, averaged across the 298 Cancer Modules, are computed through 5-fold cross-validation techniques repeated 5 times for different fixed recall levels ranging from 0.1 to 1. *S_AV _*(*Average score*), *S_NN _*(*Nearest-neighbor score*) and *S_kNN _*(*k-Nearest-neighbor score*) represent kernelized score functions. The parameter *k *of *S_kNN _*is set to 27. *Zhou *is the algorithm based on Gaussian Random Fields proposed in [31] and *GeneMANIA *its variant, while *LabelProp *is the *Label Propagation *algorithm proposed in [17].

**Figure 8:**
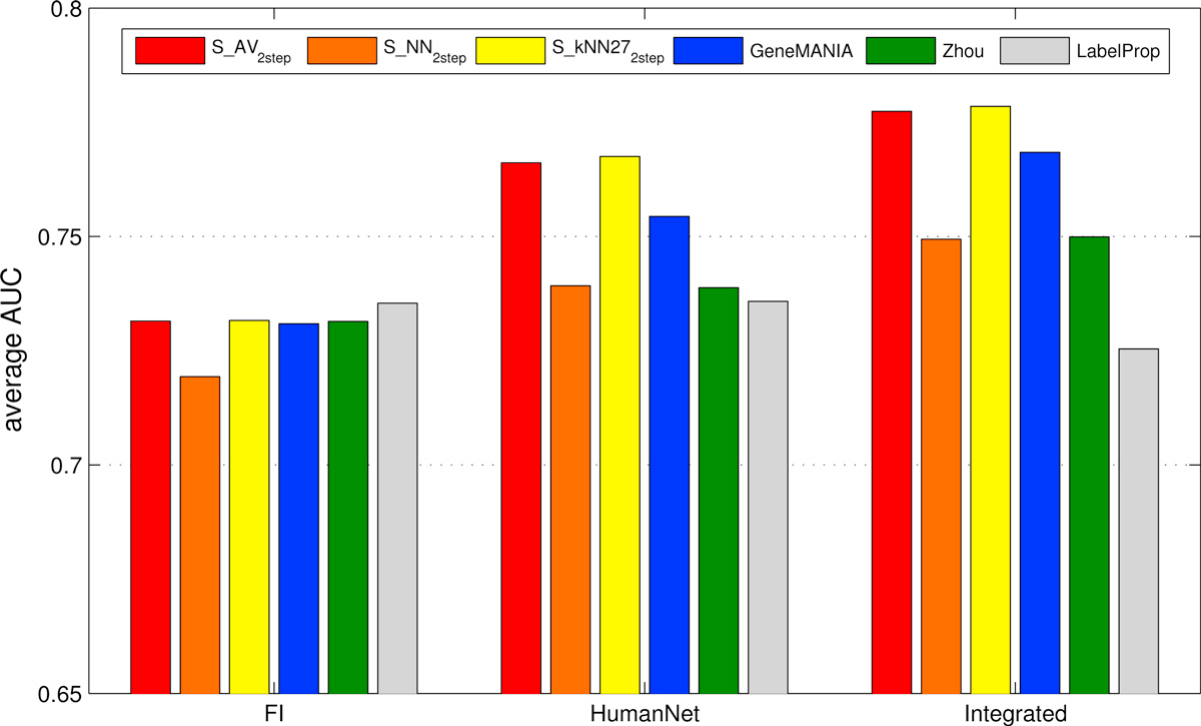
**Comparison of the AUCs (Area Under the Curve) between our proposed kernelized score functions and othe machine learning methods for gene ranking, using *FI*, *HumanNet *and integrated networks**. AUCs are averaged across all the modules and estimated through 5-fold cross-validation repeated 5 times. *S_AV _*(*Average score*), *S_NN _*(*Nearest-neighbor score*) and *S_kNN _*(*k-Nearest-neighbor score*) represent kernelized score functions. The parameter *k *of *S_kNN _*is set to 27. *Zhou *is the algorithm based on Gaussian Random Fields proposed in [31] and *GeneMANIA *its variant, while *LabelProp *is the *Label Propagation *algorithm proposed in [17].

The *FI *and *HumanNet *networks contain complementary information (see "Functional interaction networks"). We thus produced an integrated network simply by summing their adjacency matrices and we repeated our ranking experiments. Compared precision performances are depicted in Figure 7, while the averaged AUCs obtained by each method in the ranking tasks performed using the three functional networks are reported in Figure 8.

The best performing methods at low recall levels are *S_AV _*and *S_kNN_*, indicating that in the investigated ranking tasks they are the choice of election when relatively high precisions are required by the application domain (Figure 7). When we use the integrated network, the precisions of *S_AV _*and *S_kNN _*lie above (or are equals to) the ones obtained by *GeneMANIA *from 0:1 to 0:8 recall values and are slightly worse at recall 0.9 and 1. In terms of precision at low recall levels *S_AV _*outperforms *S_kNN_*: this holds until recall 0.7. It is worth noting that in this context precision at relatively low recall level is more significant, since to assess by wet-based experiments whether top ranked "false positive" genes are associated to a specific tumor, we would like to know in advance that positive predictions are actually positive (high precision), since usually wet-based experiments can be expensive in terms of costs and time. Moreover at very high recall level the precision is too low to be useful in practice (Figure 7).

According to the expectation that the information encoded by the *FI *and *HumanNet *networks are, at least in part, not overlapping, the ranking performances obtained with the integrated network are better than those obtained using either of the two component functional networks for *S_AV _*and *S_kNN_*. It should be noticed that with *GeneMANIA *we performed also a weighted combination, according to the procedures described in [32] (see Methods), but the results are statistically indistinguishable from that obtained with the simpler unweighted integration, and have been not reported in Figure 7 and 8. Indeed, the weights assigned to *FI *and *HumanNet *are approximately equal, and the resulting integrated network is very close to that obtained through the unweighted sum.

Figure 8 shows that for all but one (*LabelProp*) evaluated methods, AUC, averaged across repetitions of the experiments and all the CMs, increased after the integration of the functional interaction networks. In terms of average AUC, the best performing methods are *LabelProp *when we rank the genes using the *FI *network, and *S_AV _*and *S_kNN _*with *HumanNet *and the integrated network. With respect to the AUC, *S_kNN _*obtained, on the average, results better than the ones obtained by *S_AV _*in the test involving *HumanNet *and the integrated network. To better evaluate if the observed differences in terms of AUCs are significant, we performed a Wilcoxon signed ranks sum test by comparing the per CM AUCs averaged across the CV folds and the repetitions using the integrated network. This confirmed that *S_AV _*performs better than *GeneMANIA *(p-value: 5.864*×*10^-6^), *S_kNN _*performs better than both *GeneMANIA *(p-value: 1.162 *×*10^-7^) and than *S_AV _*(p-value: 1.332 *× *10^-6^).

To assess the potential impact of the cardinality of the CMs on the performance of the compared methods, we analyzed the precision at 0.2 recall and the AUC for CMs grouped by cardinality (Table 1). Kernelized score functions achieve the best results among the compared methods for the groups (20-100) and (101-200), that is the groups including the CMs with a low or a relatively low number of genes, independently of the considered performance measure. Moreover our proposed method obtains the best AUC also for the group (201-300). On the contrary *GeneMANIA *achieves the best results for the group including CMs with the largest number of genes, but note that *S_kNN _*achieves comparable results also in the "301 and more" group of CMs. Among the four cardinality groups the first one (20 to 100 positives) accounts for about 70% of the 298 CMs involved in our experimental setting, while each of the remaining cardinality groups covers about 10% of the 298 CMs.

**Table 1:** Compared average performances grouped by cardinality of CMs.

Precision at 0.2 recall				
***CM_group_***	***S_kNN_***	***GeneMANIA***	***Zhou***	***LabelProp***
20 to 100	**0.2040**	0.1822	0.1872	0.0534
101 to 200	**0.1851**	0.1670	0.1607	0.0173
201 to 300	0.1792	**0.1928**	0.1991	0.0342
300 and more	0.2591	**0.2620**	0.2069	0.0359

**average AUC**				
***CM_group_***	***S_kNN_***	***GeneMANIA***	***Zhou***	***LabelProp***

20 to 100	**0.7990**	0.7876	0.7779	0.7624
101 to 200	**0.7149**	0.7048	0.6773	0.6474
201 to 300	**0.7267**	0.7173	0.6804	0.6458
300 and more	0.7510	**0.7525**	0.6966	0.6213

Compared average precision at 0.2 recall and average AUC across 298 CMs grouped by cardinality (number of genes included in the CMs) obtained by 5-folds cross-validation repeated 5 times.

Summarizing, results with integrated functional interaction networks show that the combined local and global learning strategies embedded in kernelized score functions lead to significantly better results that those achieved by other compared methods. Moreover a fine tuning of the choice of the score functions and of the kernel parameters for each CM could yield to even better results.

#### Comparison of the empirical time complexity

Table 2 reports the time required by each of the compared methods for the realization of the entire experiment (5-folds CV repeated 5 times for all the 298 CMs, including pre-processing and normalization of networked data), using an Intel i7-860 2.80 GHz processor with 8 Gb of RAM. Our proposed methods are from ten to several thousands times faster than the other compared methods.

**Table 2:** Time requirements of the compared methods.

Method	*FI*	***HumanNet***	Integrated network
*S_AV_*	200	196	195
*S_NN_*	202	212	203
*S_kNN_*	391	401	400
*GeneMANIA*	1906	1981	3321
*Zhou*	62875	63005	58420
*LabelProp*	609545	610520	606420

Time required for each of the compared methods for the realization of the entire experiment (ranking of 8499 genes according to their likelihood to belong to 298 CM, 5-folds CV repeated 5 times). Times are expressed in seconds.

The proposed approach is very fast, since no model learning is required, but only a computation of scores based on kernelized distances: once the kernel matrix has been computed, the score computation has a complexity $O\left(\left|V|\cdot |V_{C}\right|\right)$, that is approximately linear when the number of "positive" nodes is largely lower than the overall number of vertices. In our experiments the number of genes in Cancer Modules is between 20 and about 600, while the number of the overall genes is larger than 8000. Hence, in this setting our algorithm is approximately linear with respect to the number of genes.

### A preliminary application to the discovery of novel genes involved in the onset and progression of cancer

Since genes associated to CMs are detectable using also data different from simple expression, we hypothesize that mining more general functional interaction networks we could extract genes that are functionally related to CMs, but whose functional interactions are lost during the construction of the expression signature. If this hypothesis is true, we expect that the top "false positive" ranked genes associated to a given CM are on the one hand functionally coherent, that is involved in the same set (or, at least, in a restricted set) of biological processes, and on the other hand pathologically coherent (that is, involved in types of cancers where the CM is activated or repressed). A thorough analysis of these topics is beyond the scope of this paper, and would require a specific study left for future research. Nevertheless in this section we present a preliminary test restricted to the CM 234 (*Bone osteoblastic module*) to show the potentialities of this approach. This choice is motivated by the fact that this CM is the only one described with a certain detail in the work of Segal and colleagues [9] with more than 19 genes, and thus is present in our experiments (we filtered out all the CMs with less than 20 genes - see subsection "Experimental set-up").

#### Evaluation of the functional coherence of the CM 234 gene ranking

The performance obtained by the compared methods in the prediction of CM 234 genes are reported in Table 3. According to the ranking obtained with *S_kNN_*, *k *= 27 (a very similar ranking has been obtained with *S_AV_*), we found that the first gene annotated in CM 234, (the bone morphogenetic protein 7, BMP7) ranked only tenth. The 9 top ranked "false positive" genes are: NPR2, COL6A3, DLX6, COL1A2, NPPB, BMP6, COL3A1, DLX2 and COL6A1, ranked in this order. To evaluate the functional coherence of this set of genes, we applied a functional profiling test of this list of 9 genes using gProfiler [34,35]. Results revealed that some of the genes in this list are associated with the GO biological process (BP) term GO:0001501, skeletal system development (p-value: 1.34*×*10^-9^), consistently to one of the gene sets, skeletal development, initially involved in the definition of CM 234. We also found a significant functional association with the GO cellular component (CC) terms GO:0005578, proteinaceous extracellular matrix (p-value: 1.13 *× *10^-5^) and GO:0030934, anchoring collagen (p-value: 6.95 *× *10^-6^). Moreover, a closer look at the members of the gene sets involved in the construction of CM 234 (see [36]) revealed that the bone morphogenetic protein 6 (BMP6) was present in the skeletal development gene set used in the construction of CM 234 but was not included in the final CM. These observations confirmed the functional coherence of these top-ranked genes, supporting the hypothesis that the proposed method is able to discover genes that are involved in the same biological processes represented by the considered expression signatures.

**Table 3:** Performance of the compared methods for the prediction of CM 234.

Method	Prec. at 0.2 recall	Prec. at 0.4 recall	average AUC
*GeneMANIA*	0.0621	0.0547	0.8701
*S_kNN_*	0.2564	0.0900	0.8527
*Zhou*	0.1219	0.0829	0.8434
*LabelProp*	0.0212	0.0395	0.7483

Average precision at 0.2 and 0.4 recall and average AUC for the CM 234 (5-folds CV repeated 5 times).

#### Evaluation of the pathological coherence of the CM 234 gene ranking

CM 234 is composed of genes involved in the proliferation and differentiation of bone-building cells [9]. The genes included in this module were found to be induced in arrays obtained from breast cancer, hepatocellular carcinoma (HCC) and nontumor hepatitis-infected liver samples [9]. Genes in this CM were also found to be repressed in subsets of HCC, in a subset of acute lymphoblastic leukemia (ALL), and in a subset of lung cancer samples.

Details about the performance of the compared methods with respect to CM 234 are presented in Table 3. This table shows the average precision at 0.2 and 0.4 recall, and the average AUC of the methods. In terms of precision at both 0.2 and 0.4 recall the kernelized score function achieve the best results, while in terms of average AUC *GeneMANIA *obtains slightly better results than the other methods.

To test the pathological coherence of the list of the 9 top ranked "false positive" genes found by *S_kNN _*(see the previous subsection), we mined the literature searching for evidences suggesting that those genes are involved in liver, breast, lung cancer or ALL. The equivalence of gene names or symbols was assessed using the information available for each gene in the Human Gene Compendium [37]. COL6A3 was recently found to be overexpressed in a study aimed at the investigation of extracellular matrix dynamics in Hepatocarcinogenesis in two mouse models [38], supporting the usefulness of data derived from more than one species in the investigated ranking tasks. The DLX gene family encodes for homeobox transcription factors involved in the control of morphogenesis and tissue homeostasis. A recent work [39] reported evidences that DLX6 is activated during metastasis formation in a breast cancer cell line. An insertion/deletion polymorphism in the 3' untranslated region of type I collagen a2 (COL1A2) was recently associated with susceptibility for HCC in a Chinese population in [40]. In a recent work [41] the authors described a molecular mechanism by which BMP6 suppresses breast cancer metastasis. Another recent work [42] reported that CpG islands in the homeobox DXL2 gene are significantly more methylated in a subtype (Luminal A) of breast tumors. A quantitative analysis focused on the study of the lung cancer cell secretome revealed that COL6A1 is a metastasis-associated protein [43].

The 9 top ranked "false positive" genes predicted by the *Zhou *method are NPPB, NPPC, NPPA, COL6A3, FN1, COL3A1, NPR2, COL1A2 and FURIN (ranked in this order). The COL6A3, COL3A1, COL1A2 and NPR2 genes are also present in the top ranked prediction of our proposed method. The natriuretic peptide precursor B (NPPB) has been recently investigated as potential biomarker in lung cancer [44]. The C-natriuretic peptide NPPC can significantly decrease the number of small-cell lung cancer cells as demonstrated in [45]. It was not possible to found supporting literature for the association of NPPA with the tumor types in which CM234 was found to be activated or deactivated by Segal and colleagues. In [46] FN1 was sought to be of prognostic value using a univariate analysis of gene expression. FN1 was also bound to be a potential biomarker for hepatocellular carcinomas in [47]. FURIN is involved in the modulation of the activity of the membrane type-1 matrix metalloproteinase (MT1-MMP), an enzyme for which a protumorigenic action has been recently observed [48] in breast cancer cells.

The 9 top ranked "false positive" genes predicted by *GeneMANIA *are SFTPC, NPPB, CHRDL2, NPPC, NPPA, DLX6, GALNT3, GLRB and DLX1 (ranked in this order). Of these genes three (NPPA, NPPB and NPPC) are also present in the list of top ranked false positives predicted by the *Zhou *method while the DLX6 gene was also predicted as top ranked false positive by our proposed method. Quite interestingly kernelized score functions predicted as top ranked false positives two members of the DLX genes family (DLX2 and DLX6), while *GeneMANIA *predicted as false positive another member of the family (DLX1). Among the false positives predicted only by *GeneMANIA*, we observe that GALNT3 is a target of the ERBB2 oncogene in breast cancer [49].

The 9 top ranked "false positive: genes predicted by the *LabelProp *method are GRB2, ACTB, PRKACA, SP1, MAPK1, HSP90AA1, HSPA8, MAPK14 and SRC (ranked in this order). In this case we found a less strict evidence of association with the tumor types related to CM 234. Moreover there is no overlap with "false positive" top ranked genes of the other methods. This is not surprising since this method behaves poorly with respect to both precision at fixed recall and AUC (Table 3).

Summarizing, three of the considered methods (kernelized score functions, *GeneMANIA *and *Zhou*) are able to detect novel genes associated to cancer types related to CM 234, but not included yet in CM 234 itself. These results show that by exploiting functional interaction data not limited to gene expression data, our proposed kernelized score functions and other state-of-the-art gene ranking network-based methods could be in perspective applied to discover novel genes involved in different cancer types related to specific CMs, thus mitigating a serious problem affecting expression signature based approaches: the difficulty in placing these signatures in a wider biological context.

## Conclusions

In this paper we applied state-of-the-art semi-supervised machine learning methods to rank genes according to their likelihood to belong to specific CMs, using gene networks constructed from several sources of functional interaction data, such as Reactome and other curated pathways databases, physical protein-protein interactions, proteins domain-domain interactions, protein interactions obtained via biomedical text mining and Gene Ontology annotations, and functional interactions derived from yeast, fly and worm by means of a comparative genomics approach.

Results show that using these integrated networks we can successfully predict CMs defined mainly with expression signatures obtained from gene expression data profiling. In particular the integration of *FI *and *HumanNet *networks leads to the best results, independently of the method applied.

Our proposed kernelized score functions compare favorably to state-of-the-art semi-supervised machine learning methods, both in terms of average AUC and precision at a fixed recall, at least for recall levels lower than 0.7, where a meaningful precision can be achieved in this difficult gene ranking task.

The substantial linearity of the proposed score functions (that holds when the number of "positive" genes is largely lower than the overall number of genes) assures the scalability and applicability of the method to very large gene networks, as shown also by its empirical computational time, significantly lower with respect to the other compared methods.

Moreover, the analysis of the ranking results obtained for the "Bone osteoblastic module" (CM 234), shows that our approach is able to detect genes involved in several types of cancer related to the same Cancer Module, but not necessarily included in the Cancer Module itself. These results show the potentiality of our proposed methods for the discovery of novel genes involved in the onset and progression of tumors related to CMs, and a full genome study, extended to all CMs, is left for future research.

Another possible research line could be the study of learning strategies able to explicitly take into account the similarities between different CMs. Indeed learning a CM could be useful to better learn other related CMs and some kind of knowledge transfer [29] or also multi-task learning strategies could be explored in this context.

## Competing interests

The authors declare that they have no competing interests.

## Authors' contributions

Authors equally contributed to this work.
